# Genetic control of tracheid properties in Norway spruce wood

**DOI:** 10.1038/s41598-020-72586-3

**Published:** 2020-10-22

**Authors:** J. Baison, Linghua Zhou, Nils Forsberg, Tommy Mörling, Thomas Grahn, Lars Olsson, Bo Karlsson, Harry X. Wu, Ewa J. Mellerowicz, Sven-Olof Lundqvist, María Rosario García-Gil

**Affiliations:** 1 Department of Forest Genetics and Plant Physiology, Umeå Plant Science Centre, Swedish University of Agricultural Science, Umeå, Sweden; 2 RISE Bioeconomy, Box 5604, 114 86 Stockholm, Sweden; 3 IIC, Rosenlundsgatan 48B, 11863 Stockholm, Sweden; 4 Skogforsk, Ekebo 2250, 268 90 Svalov, Sweden

**Keywords:** Agricultural genetics, Genetic association study, Genome-wide association studies, Genetics, Quantitative trait

## Abstract

Through the use of genome-wide association studies (GWAS) mapping it is possible to establish the genetic basis of phenotypic trait variation. Our GWAS study presents the first such effort in Norway spruce (*Picea abies* (L). Karst.) for the traits related to wood tracheid characteristics. The study employed an exome capture genotyping approach that generated 178 101 Single Nucleotide Polymorphisms (SNPs) from 40 018 probes within a population of 517 Norway spruce mother trees. We applied a least absolute shrinkage and selection operator (LASSO) based association mapping method using a functional multi-locus mapping approach, with a stability selection probability method as the hypothesis testing approach to determine significant Quantitative Trait Loci (QTLs). The analysis has provided 30 significant associations, the majority of which show specific expression in wood-forming tissues or high ubiquitous expression, potentially controlling tracheids dimensions, their cell wall thickness and microfibril angle. Among the most promising candidates based on our results and prior information for other species are: *Picea abies BIG GRAIN 2* (*PabBG2)* with a predicted function in auxin transport and sensitivity, and *MA_373300g0010* encoding a protein similar to wall-associated receptor kinases, which were both associated with cell wall thickness. The results demonstrate feasibility of GWAS to identify novel candidate genes controlling industrially-relevant tracheid traits in Norway spruce.

## Introduction

Norway spruce is considered to be one of the most important multipurpose species. Its wood provides various solid wood products as well as pulp and paper products. It is considered one of the best raw-materials for the production of mechanical pulp for many types of paper grades^1^. The properties of the tracheids have large influences on the quality of the final products, and also on process economy andì sustainability, for solid wood as well as fibre-based products^2^. Tracheid morphology and cell wall structure influence the flexibility of wood and fibres, interactions among fibres, as well as the mechanical, physical and optical properties of the end-products^3^. Consequently, identifying the genetic background of different tracheid traits as a basis for breeding may bring benefits for both industry and society. Several papers have reported the phenotypic correlations, between tracheid cross-sectional dimensions and wood traits such as density in conifers^4–6^. A study of Norway spruce felled in the winter of 1989/1990 in central Sweden, found tracheid length dependent on the logarithm of cambial age and growth ring width, with density dependent on latewood percentage. Similar models for the influence of cambial age and ring width have been presented for tracheid length, width and wall thickness models of Norway spruce, Sitka spruce, Scots pine and loblolly pine^7^. Such results have indicated that changes in growth conditions over time acting mainly through crown development, will have an influence on wood structure development in Norway spruce^6^. However, these reports paid very little regard to the underlaying genetic factors influencing these phenotypes. Therefore, the dissection of the genetics impacting these relationships and the variations observed in tracheid properties will be of great value to any tree breeding program.

Various long-term breeding programmes for the species are already being pursued with the goal to identify genotypes with high productivity and wood quality^8^. Wood density and microfibril angle (MFA) are key indicators of wood quality as they influence strength and dimensional stability of solid wood^9^. However, combining productivity with wood quality is problematic due to negative genetic correlations between these traits^10^. One of the tools helping to understand these genetically complex variations in forest trees is the integration of extensive genetic and phenotypic data in order to discern the genetics underlying these traits^11–13^. Hence, knowing the genetic control of these variations, may lead to optimal breeding strategies for the improvement of both growth and wood quality traits.

With genomic resources now available, a large array of molecular markers has been available for the studying and understanding of complex traits. The majoritiy of these traits are known to be predominantly polygenic in nature, and affected by environmental effects^14^, hence the need to utilize techniques that target the whole genome^15^. The availability of an array of genomic resources has led to the reliable identification of Quantitative Trait Loci (QTLs), which in conifers are traditionally detected using suitable segregating populations such as, full- or half-sib progenies. More recently, GWAS, also known as Linkage Disequilibrium (LD) mapping, has been applied as an alternative approach of QTL detection from traditional pedigree-based mapping studies. GWAS accounts for historical recombination events in the natural population as compared to those observed in a pedigree based QTL mapping^16^. When confounding factors are taken into consideration, LD mapping provides greater resolution than pedigree studies, since it utilizes markers in strong LD with putative causative genomic regions^17^.

Many coniferous species are characterized by an outcrossing mating system and large population sizes which lead to a rapid LD decay within the genomes and low inbreeding coefficient^16^. However, rapid and heterogenous decay in conifer LD^18^ can be a source of concern as proximal markers can be completely unlinked and therefore offer no predictive power to the quantitative trait that may be residing physically close^19^. Together LD heterogeneity, population structure^20^, epistasis and Genotype x Environment interactions (GxE)^21^ are factors that if not carefully controlled can negatively impact QTL identification. The utilization of LD mapping in the dissection of genetic backgrounds underpinning complex traits has been shown in several systems, for example, complex solid wood properties in Norway spruce^22^, white spruce^23^ and *Eucalyptus*^19^, and detecting genes underlying various traits including ecological adaptations in *Populus*^24–27^. The dissection of these complex traits can benefit from the application of mathematical functions that account for the year-to-year variation across annual growth rings. The development of mathematical methods for the analysis of dynamic data has made it possible to develop functional mapping approaches^28,29^ that firstly model the phenotypes using curve-fitting methods and then utilize the parameters describing the curve (latent traits) for independent association analysis^30,31^.

GWAS can also increase our knowledge on molecular processes controlling tracheid traits. Presently the majority of breeding programs have focused on the easy to measure phenotypic traits such as volume, straightness, disease resistance and spiral grain. Due to cost and time of measurement of traits related to tracheid dimensions most programs have not been able to select and advance such traits using marker assisted breeding^32^.Therefore, this study is novel in that it is, to our knowledge, one of the first to tackle the issue of dissecting the genetic background to tracheid properties in a conifer species. With the exception of a single study conducted in *Arabidopsis thaliana*, as a model system, for traits controlling fibre length^33^, the majority of the studies related to tree fibre related traits have focused on mostly microfibril angle genetics^34–37^. Hence our study seeks to form the bases upon which, the dissection of the genetic backgrounds to more complex and expensive traits, such as, tracheid dimensions can be investigated. Such traits are to a large extent determined by the genes acting during wood development^38,39^. Tracheid traits can also be regulated non-cell-autonomously by processes that take place in other organs and tissues. For example, the activity of the shoot apical meristem determines the availability of auxin in the cambium and developing wood^40,41^, whereas the photosynthetic activity in the needles influences the availability of sucrose for wood biosynthesis^25^. Therefore, combining the knowledge of candidate genes with their expression analysis will give more insights to the biological processes shaping tracheids.

The major goal of this study was to identify causative allelic effects of genomic regions contributing to wood tracheid traits using LD mapping on exome sequence capture data. Due to the large size of the Norway spruce genome (20 Gb) and its highly repetitive nature, it presents a challenge to use whole genome re-sequencing approaches for the development of molecular markers. Approaches aimed at reducing these genome complexities, especially by either eliminating or drastically reducing the repetitive sequences have been developed^42^. These approaches are referred to as reduced representation approaches as there are used as proxies for whole genomic sequencing. In this study, we have used exome capture, aiming at maximizing the capture of exonic regions of the genome only, thereby increasing the coverage and depth of genic sequence in our variant detection study. The analysis provided 26 mostly novel candidate genes for regulation of various tracheid traits, which, along with their expression patterns, give new insights to the tracheid traits determination, and offer key markers for early genetic selection in Norway spruce breeding.

## Materials and methods

### Association mapping population

The association mapping population, phenotypic data and statistical analysis are described in Chen et al*.*^10^ and Hayatgheibi et al*.*^43^. Briefly, the mapping population for the association mapping population constisted of two progeny trails established 1990 in Southern Sweden: (S21F9021146 aka F1146 (trial1) and S21F9021147 aka F1147 (trial2)), composed of 1373 and 1375 half-sib families. A randomized incomplete block design with single-tree plots was employed for both trials. From the trials, 517 families in 112 provenances were selected for use in the investigation of wood tracheid properties. Wood increment cores with diameter of 12 mm were collected at breast height (1.3 m) from six trees from each of the selected families of each trial. A total of 5618 trees were sampled: 2973 trees from trial F1146 and 2645 from F1147.

### Phenotypic data generation

The radial variations of growth, wood and tracheid attributes from pith to bark were analysed using the SilviScan instrument^44^ at Innventia, now RISE Bioeconomy, Stockholm, Sweden. SilviScan is an instrument for efficient measurement of radial variations in a multitude of properties from the same sample with high spatial resolution. High precision sample strips from pith to bark were produced from the increment cores and automatically scanned for radial variations in cross-sectional tracheid widths with a video microscope combined with image analysis, in wood density with X-ray transmission and in structural orientations with X-ray diffraction. From these data, information on radial variations of further traits were derived, such as wall thickness, coarseness and MFA of tracheids, and stiffness of wood (MOE). The locations of the annual rings were identified, as well as of their compartments of earlywood (EW), transitionwood (TW) and latewood (LW), using the “20–80 density” definition^45^, established for use in different types of studies^46–48^. Averages for all rings and their compartments were calculated for the traits and organised to be ready for use in continued genetic evaluations, such as the work on solid wood traits^10^, on tracheid traits^49^ and for wood traits^22^, genomic selection^50^ and influences of age and weather^51^. The traits addressed in the current work are listed in Table 1.

**Table 1:** List of the traits, their abbreviations and measurement unit.

Trait	Abbreviation	Unit
**Radial tracheid width (TWr)**		
Ring	TWr_Ring_	μm
Earlywood	TWr_EW_	μm
Transitionwood	TWr_TW_	μm
Latewood	TWr_LW_	μm
**Tangential tracheid width (TWt)**		
Ring	TWt_Ring_	μm
Earlywood	TWt_EW_	μm
Transitionwood	TWt_TW_	μm
Latewood	TWt_LW_	μm
**Wall thickness (WT)**		
Ring	WT_Ring_	μm
Earlywood	WT_EW_	μm
Transitionwood	WT_TW_	μm
Latewood	WT_LW_	μm
**Coarseness (C)**		
Ring	C_Ring_	mg/m
Earlywood	C_EW_	mg/m
Transitionwood	C_TW_	mg/m
Latewood	C_LW_	mg/m
**Microfibril angle (MFA)**		
Ring	MFA_Ring_	Degrees
Corewood	MFA_CORE_	Degrees
Outerwood	MFA_OUTER_	Degrees
Transition age (cambial)	MFA_TA_	Year

For MFA, central peak regression mathematical functions were fitted to describe the MFA variation from juvenile towards mature wood, using procedures presented by Hayatgheibi et al.^43^, including also pre-processing of the data for removal of outliers. A threshold value of MFA 20° for the fitted curves was chosen to define an age up to to which an inner core of wood with inferior timber properties occurred, here named the transition age MFA_TA_^43^. From anatomical perspective, a threshold of 20° is on the high side, emphasizing a core of pronounced juvenility. We have decided to stay with this threshold level, because for the young trees investigated, the fitted curves for quite a few trees would not pass a low treshold, and they would have had to be discarded from the analysis. Thus, it works better for ranking. The averages of MFA for wood inside and outside this limit were calculated, MFA_CORE_ and MFA_OUTER_. This provided three latent traits for MFA.

#### Exome capture analysis

DNA extraction, variant detection and annotation and population structure on the genomic data utilized in this study was previously described^22^. Total genomic DNA from 517 half-sib individuals was extracted using the Qiagen Plant DNA extraction kit (Qiagen, Hilden, Germany). DNA was extracted from buds, when present, or from young needles, when buds were absent. DNA quantification was performed using the Qubit ds DNA Broad Range (BR) Assay Kit (Oregon, USA). DNA from randomly selected individuals was then electrophoresed on a 2% agarose gel. Probe design and evaluation is described in Vidalis et al.^52^. In breif, the exome capture method was implemented by the probe design that was based on a combination of sequenced genomic DNA, predicted gene annotations and de novo transcript assemblies. Exome capture was based upon the use of targeted oligonucleotides that bind to complementary genomic sequences. Sequencing was performed at Rapid Genomics, USA, using the Illumina sequencing platform. Sequence capture with average depth of 15× coverage was performed using the 40,018 diploid probes previously designed and evaluated for Norway spruce. Illumina sequencing compatible libraries were amplified with 14 cycles of PCR and the probes were then hybridized to a pool comprising 500 ng of 8 equimolarly combined libraries following Agilent’s SureSelect Target Enrichment System (Agilent Technologies). These enriched libraries were then sequenced to an average depth of 15× using an Illumina HiSeq 2500 (San Diego, USA) on the 2 × 100 bp sequencing mode at Rapid Genomics, USA.

Raw reads were mapped against the *P. abies* reference genome v1.0 using BWA-MEM^53^. SAMTools v.1.2^54^ and Picard (https://broadinstitute.github.io/picard) were used for sorting and marking of PCR duplicates. Variant calling was performed using GATK HaplotypeCaller v.3.6 as per the best practices protocol^55^ in gVCF output format (see https://www.broadinstitute.org/gatk/guide/best-practices for more information about GATK best practices). Samples were then merged into batches of ~ 200 before all 517 samples were jointly called.

GATK based Variant Quality Score Recalibration (VQSR) method was performed in order to avoid the use of hard filtering for exome/sequence capture data. For the VQSR analysis, two datasets were created: a training file and an input file. The training dataset was derived from a Norway spruce genetic mapping population with known segregating loci. The training dataset was designated as true SNPs and assigned a prior value of 12.0. The input file was derived from the raw sequence data using the above mentioned GATK’s best practices with the following parameters: extended probe coordinates by +100 excluding INDELS, excluding the LowQual sites, and keeping only bi-allelic sites. The annotation parameters QualByDepth (QD), MappingQuality (MQ) and BaseQRankSum, with tranches 100, 99.9, 99.0 and 90.0, were then applied to the two files for the determination of the good versus bad variant annotation profiles. After obtaining a VQSR for all raw data variant sites, the recalibration was applied to filter the raw variants. The SNP trimming and cleaning involved the removal of any SNP with MAF and “missingness” of < 0.05 and > 20%, respectively. These parameters were filtered out using VCFTools^56^. The resultant SNPs were annotated using default parameters for snpEff 4^57^. Ensembl general feature format (GTF, gene sets) information was utilized to build *P. abies* 1.0 snpEff database.

#### GWAS LASSO

Latent traits expressing how the traits developed with age were calculated in two steps. First, a breeding value approach was applied to refine data from influences not directly related to the genes, such as site and block effects. For this purpose, breeding values were estimated (EBV) for each annual ring separately (cambial age), reducing site and block effects, but also the time trajectories, which were reconstructed as a final step by adding back the averages at each age. The variance and covariance components were estimated using ASREML 4.0 as described in Chen et al.^10^. The EBVs at each cambial age were estimated using univariate, bivariate or multivariate mixed linear models in order to select the optimal model for each trait, based on a compromise of model fit and complexity. Akaike Information Criteria (AIC) was used to determine the fitness of different models. This resulted in use of a univariate linear mixed model for joint-site analysis as the bases for the analyses of all traits:

1 $$Y_{ijkl} = u + {\text{S}}_{i} + {\text{B}}_{j\left( i \right)} + {\text{F}}_{k} + {\text{SF}}_{ik} + {\text{e}}_{ijkl}$$

where ${\text{Y}}_{ijkl}$ is the observation on the *l*th tree from the *k*th family in ${\text{j}}$th block within the *i*th site, *u* is the general mean, ${\text{S}}_{i}$ and ${\text{B}}_{j\left( i \right)}$ are the fixed effects of the *i*th site and the *j*th block within the ${\text{i}}$th site, respectively, ${\text{F}}_{k}$ and ${\text{SF}}_{ik}$ are the random effects of the *k*th family and the random interactive effect of the *i*th site and *k*th family, respectively, ${\text{e}}_{ijkl}$ is the random residual effect.

**Figure 1:**
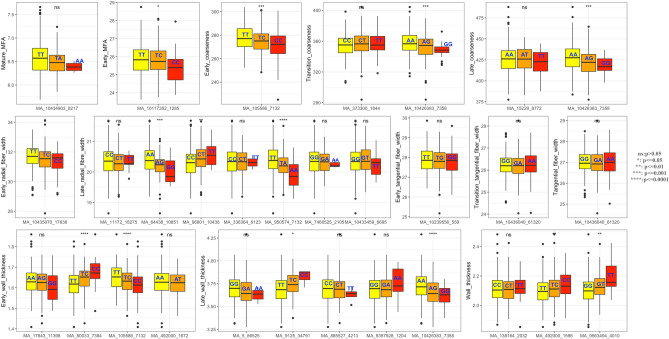
Box plot of the estimated genotypic effects for all significant associations identified in the study. The middle line represents the median value of the phenotype with that of the genotype. Upper and lower bounds of the box are the 25% (Q1) and 75% (Q3) quantile. Whiskers are Q1-1.5*Interquartile range (IQR) and Q3 + 1.5*IQR, therefore the outliers are values outside the range (Q1-1.5*IQR to Q3 + 1.5*IQ. Yellow, orange and red colored boxplots indicate the genotypic classes per SNP. Significant level were obtained by Kruskal–wallis test^68^.

For the tracheid dimension and coarseness traits, linear splines with multiple knots were fitted to the EBV refined time trajectories against cambial age (annual ring number) (Fig. 1), generally defined as follows:

2 $$y(t) = \beta_{0} + \beta_{1} t + \beta_{2} (t - K_{1} )_{ + } + \beta_{3} (t - K_{2} )_{ + } + \cdots + \beta_{1 + m} (t - K_{m} )_{+} ,$$

This is a continuous curve starting at the intercept β_0_, with linear segments between the knots at *t* = *K*_i_ (*i* = 1,…,*m*; *K*_1_ < *K*_2_… < *K*_*m*_), segments with slopes defined by the β_1_ to β_1+m_ parameters, where β_i_ = 0 if t < K_i-1_. The knots are thus reflecting transitions between phases of different slopes in the development of the traits, and at each knot, the slope is changed according to the β of the next segment. Therefore, the times when the knots occur have to be properly defined in order to provide accurate descriptions of the data under investigation, and also their numbers in order to avoid overadaptation to data^31,58^. We found use of two knots the most suitable for tracheid dimension traits across the time intervals investigated. Hence, the linear spline model used was defined as:

3 $$y(t) = \beta_{0} + \beta_{1} t + \beta_{2} (t - K_{1} )_{+} + \beta_{3} (t - K_{2} )_{ + } + \varepsilon_{i} (t),\quad \varepsilon_{i} (t)\mathop \sim \limits^{\text{i.i.d.}} {\text{N}}(0,\sigma^{2}).$$

In a first analysis, fixed values of K_1_ and K_2_ were adapted for each trait. Then, the intercept *β*_0_, and the slope parameters *β*_1_, *β*_2_ and *β*_3_ were estimated for each tree by standard least squares^59^. The four estimates were used as the latent trait in the subsequent QTL analysis conducted in R-studio^60^, and then analysed using the LASSO model in order to identify SNPs showing significant associations to the traits.

The LASSO model as described by Li et al.^58^, was applied to all latent traits for the detection of QTLs.

The LASSO model:

4 $$\mathop {\min}\limits_{{(\alpha_{0} ,\alpha_{j} )}} \frac{1}{2n}\sum\limits_{i = 1}^{n} {(y_{i} - \alpha_{0} - \sum\limits_{j = 1}^{p} {x_{ij} \alpha_{j} } )^{2} } + \lambda \sum\limits_{j = 1}^{p} {|\alpha_{j} |,}$$

where *y*_*i*_ is the phenotypic value of an individual *i* (*i* = 1,…,*n*; *n* is the total number of individuals) for the latent trait, *α*_0_ is the population mean parameter, *x*_*ij*_ is the genotypic value of individual *i* and marker *j* coded as 0, 1 and 2 for three marker genotypes AA, AB and BB, respectively, *α*_*j*_ is the effect of marker *j* (*i* = 1,…,*n*; *n* is the total number of markers), and *λ* (> 0) is a shrinkage tuning parameter.

Stability selection probability (SSP) of each SNP was applied as a way to control the false discovery rate and determine significant SNPs^58,61,62^. For a marker to be declared significant, a SSP inclusion ratio (Frequency) was used with an inclusion frequency of at least 0.52 for all traits. This frequency inferred that the expected number of falsely selected markers was less than one (1), according to the formula of Buhlmann et al.^63^. Population structure was accounted for in all analyses by including the first five principal components based on the genotype data as covariates into the model. The LASSO regression has a limitation in that it might over-shrink the effect size of SNPs due to the use of a single tuning parameter for all the regression parameters^64^. The consequence is that the LASSO might significantly under-estimate the proportion of phenotypic variation (PVE) explained by a SNP^65^. To improve this, an adaptive LASSO approach^64^ was used alternatively to evaluate the PVE of a QTL (Methods S4):

In brief, estimated breeding values (EBV) were computed for each annual ring by cambial age to reduce site and block effects (see Chen et al. 2014). In a second step, linear splines were applied to reconstruct time trajectories based on annual ring EBV. Fix age values for two knots were determined, as the intercept and slope parameters, the latent traits, were fitted to the EBV describing the shape of the time trajectories of each individual tree.

#### Candidate gene mining

To assess putative functionality of SNPs with significant associations, gene ontology and network analysis of putative genes and their associated orthologs was performed against the NorWood v1.0 database (https://norwood.congenie.org69) hosted by ConGenIE (https://congenie.org/). After the identification of the QTL, the Norway spruce contigs linked to the significant SNPs were extracted from the web based database congenie (congenie.org/blast). The complete Norway spruce contigs that harboured the QTLs that were not annotated in the ConGenIE were used to perform a nucleotide BLAST (Blastn) search, using the option for only highly similar sequences (megablast) in the National Center for Biotechnology Information (NCBI) nucleotide collection database (https://blast.ncbi.nlm.nih.gov/Blast.cgi?).

## Results and discussion

### Trait trajectories

For traits with complex time/age trajectories, the application of functional mapping enables an aggregated analysis of temporal trends^30^. The ring MFA initially decreased from an average across the trees of about 30° at the pith and stabilized after reaching a cambial age of about 10 years at an average of 10°–12°^67^. The adapted central peak curves combined with the threshold at 20° resulted in an average of five years for MFA_TA_, defining the inner core of lower quality timber with AM performed for the latent traits of MFA_CORE_ and MFA_OUTER_.

For all the other tracheid phenotypes: wall thickness, radial tracheid width, tangential tracheid width and coarseness, family means of β_0_ (intercept) and β_1_ to β_3_ (effects of knot 1 to 3, see Baison et al., 2019)^22^ were implemented in the association mapping. Candidate gene loci were identified for MFA_CORE_, MFA_OUTER_ and MFA_TA_, and for the intercepts β_0,_ of the tracheid dimensions and coarseness of rings, EW, TW and LW.

### Genetic associations detected and modes of gene action

A total of 30 significant associations were detected across the 18 traits with fraction of phenotypic variances being explained (PVE) ranging between 0.01 to 3.79% (Table 2), using an Stability selection probability (SSP) minimum inclusion frequency of 0.52. Seven of the 30 marker trait associations for which dominance and additive effects could be calculated were consistent with partially to fully dominant effects (0.50 <|*d/a*|< 1.25). The remaining 23 markers were all determined to have an additive (|*d/a*|< 0.50) mode of inheritance (Table 2). Three SNPs MA_10436040g0010_171180, MA_105586g0010_65505 and MA_10426383g0010_135796, were significantly associated *across* and *within* several traits, with all the modes of gene action being additive for the marker-trait interactions for the three SNPs (Table 2; Fig. 1).

**Table 2:** Phenotype, QTL position, allele frequencies and modes of allele inheritance.

Phenotype	QTL^1^	SNP^2^	Region, Feature	Alleles (0/1)	Inclusion frequency	PVE (*H*^*2*^_*QTL*_) (%)	2a^3^	d^4^	d/a
MFA_CORE_	129,716	MA_10117352g0010_1285	Synonymous	T/C	0.548	1.47	0.403	0.168	0.836
MFA_OUTER_	165,836	MA_10434903g0010_8217	Intron variant	T/A	0.600	1.03	0.190	− 0.002	− 0.025
TWr_EW_	166,535	MA_10435070g0010_17636	Missense	T/C	0.627	3.16	0.349	− 0.053	− 0.304
TWr_LW_	95,509	MA_336364g0010_6123	Missense	C/T	0.665	0.05	0.007	− 0.039	− 11.489
	12,016	MA_11172g0010_18275	Splice variant	A/G	0.579	0.01	0.051	− 0.078	− 3.058
	160,388	MA_10433459g0010_6695	Upstream	G/T	0.604	1.72	0.118	0.042	0.701
	44,384	MA_64438g0010_10851	Missense	A/C	0.546	0.03	0.395	0.024	0.123
	59,913	MA_96801g0010_10438	Synonymous	C/T	0.547	0.10	0.212	0.022	0.211
	116,013	MA_950574g0010_7132	Upstream	T/A	0.558	0.05	0.451	0.004	0.017
	120,168	MA_7460525g0010_2105	Upstream	G/A	0.561	2.27	0.140	0.045	0.642
TWt_EW_	131,776	MA_10239556g0010_	Upstream	T/G	0.633	1.80	0.072	0.002	0.069
TWt_TW_	**171,180**	MA_10436040g0010_61320	Upstream	G/A	0.661	2.13	0.003	− 0.039	− 24.638
TWt_Ring_	**171,180**	MA_10436040g0010_61320	Upstream	G/A	0.566	3.79	0.019	− 0.046	− 4.646
WT_EW_	51,296	MA_80033g0010_7384	Missense	T/C	0.541	0.01	0.047	0.0007	0.033
	**65,505**	MA_105586g0010_7132	Missense	T/C	0.536	0.10	0.032	− 0.002	− 0.114
	19,482	MA_17843g0010_11398	Missense	A/G	0.546	0.01	0.021	0.010	0.976
	**103,329**	MA_492000g0010_1672	Upstream	A/T	0.582	0.10	0.032	− 0.021	− 1.277
WT_LW_	1	MA_5g0010_86525	Upstream	G/A	0.876	0.01	0.046	0.008	0.356
	9,848	MA_9125g0010_34791	Upstream	T/C	0.64	0.10	0.053	0.004	0.131
	112,677	MA_885527g0010_4213	Upstream	C/T	0.795	0.02	0.059	0.018	0.591
	126,271	MA_9357928g0010_1204	Upstream	G/A	0.567	0.02	0.060	− 0.024	− 0.809
	**135,796**	MA_10426383g0010_7358	Synonymous	A/G	0.61	1.57	0.097	− 0.0007	− 0.015
WT_Ring_	103,326	MA_492000g0010_1596	Synonymous	T/C	0.726	1.78	0.038	− 0.003	− 0.163
	127,327	MA_9563494g0010_4010	Missense	G/T	0.509	0.01	0.084	− 0.019	− 0.464
	78,937	MA_138164g0010_2032	Downstream	C/T	0.529	1.25	0.007	0.001	0.442
C_EW_	**65,505**	MA_105586g0010_7132	Missense	T/C	0.633	2.08	5.481	− 0.238	− 0.087
C_TW_	96,993	MA_373300g0010_1844	Upstream	C/T	0.559	3.62	1.905	0.791	0.831
	**135,796**	MA_10426383g0010_7358	Synonymous	A/G	0.53	3.25	9.214	0.533	0.116
C_LW_	16,320	MA_15229g0010_	Upstream	A/T	0.512	0.78	4.120	1.448	0.703
	**135,796**	MA_10426383g0010_7358	Synonymous	A/G	0.635	1.40	9.434	0.146	0.031

^1^Bold QTLs indicate associations that have been detected *across* and *within* traits.

^2^SNP: The SNP name is composed of contig (MA_number) and SNP position on contig. As an example, the SNP MA_10117352g0010_1285 is located on contig MA_10117352 at position 1,285 bp. QTL is the value extracted after the Stability selection probability (SSP) has been performed to indicate the significant associations. This value then points to the significant SNP.

^3^Calculated as the difference between the phenotype means observed within each homozygous class (2a =|G_BB_-G_bb_|, where G_ij_ is the trait mean in the *ij*th genotype class).

^4^Calculated as the difference between the phenotypic mean observed within the heterozygous class and the average phenotypic mean across both homozygous classes [d = G_Bb_-0.5(G_BB_ + G_bb_), where G*ij* is the trait mean in the *ij*th genotypic class].

### Genetic associations and genes of interest

**Figure 2:**
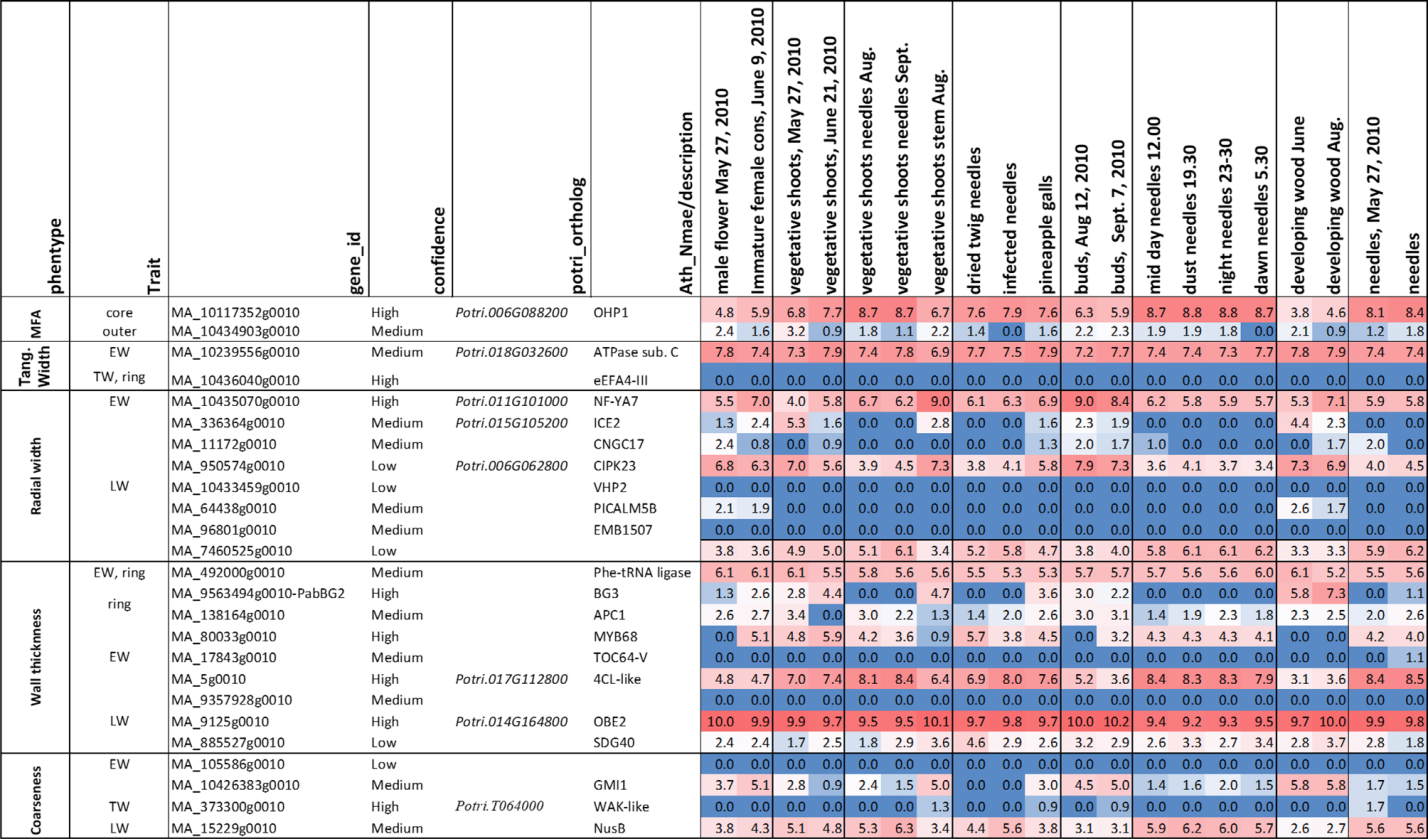
The heatmap showing levels of the variance stabilized transformed expression values (VST values) of spruce candidate genes in different organs and tissues based on ExAtlas data^74^ available at https://congenie.org. (Microsoft excel).

Two of the associations detected for MFA were intron variants MA_10434903g0010_8217 and MA_10117352g0010_1285. MA_10434903g0010_8217 was associated with MFA_OUTER_ explaining 1.03% of the PVE. MA_10117352g0010_1285, a synonymous variant explaining 1.47% PVE associated with MFA_OUTER,_ occurred within gene MA_10117352g0010 homologous to *Arabidopsis* ONE HELIX PROTEIN (OHP). The gene is highly expressed especially in needles and shoots in spruce (Fig. 2). OHPs have been reported to be constitutively expressed and essential for photosynthesis in Arabidopsis, with mutants exhibiting severe growth defects^69^.

Associations for radial tracheid widths were detected in earlywood and latewood. TWr_EW_ was associated with a single missense SNP (MA_10435070g0010_17636) explaining 3.16% of the PVE and occurred within a gene encoding nuclear transcription factor Y subunit A-7 (NF-YA7) (Table S1). NF-Y is a multimer complex binding CCAAT box in the promoter regions of many genes, and has multiple biological functions including growth regulation, cell size regulation, and responses to abiotic stresses^70,71^, including nitrogen deficiency in *Arabidopsis*^72^. The overexpression of the NF-YAs has been shown to stimulate growth during low nitrogen and phosphorous availability^73^. This gene is ubiquitously, highly expressed in shoots and buds of spruce, indicating its important function in this species (Fig. 2).

**Figure 3:**
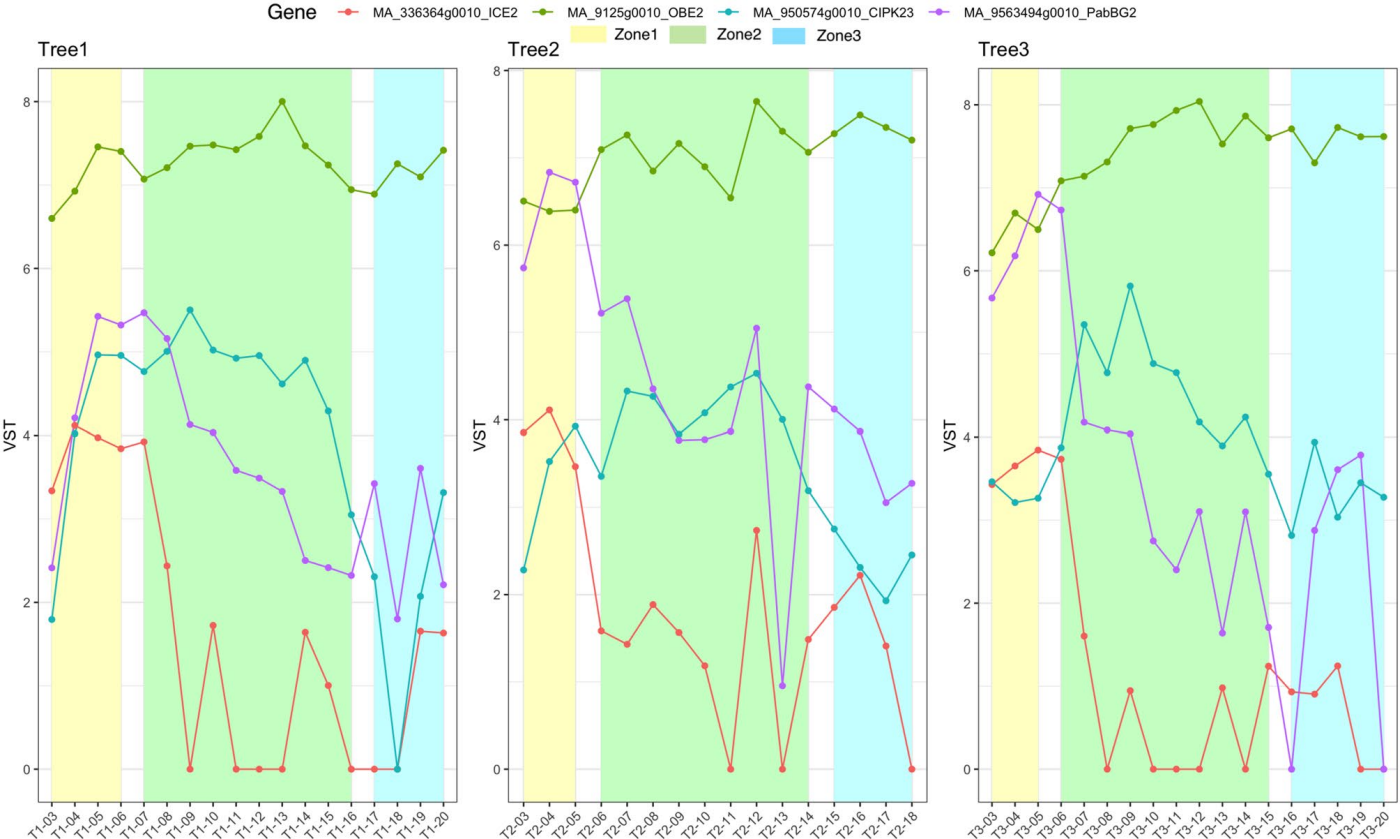
Expression (Variance stabilized transformed expression values) profiles of selected candidate genes in wood developing tissues of sections through developing wood zones, phloem to mature xylem of spruce based on NorWood dataset (https://norwood.congenie.org/norwood-v1.0/^66^). Expression profiles of three trees sampled during the peak of wood formation in the summer are shown. The X-axis shows numbers of consecutive tangential sections through the developing wood zones. The zone numbers corresponding to: (i) cambium-radial expansion zone, (ii) secondary wall formation zone, and (iii) mature zone are shown above the graphs for each treey^68^.

TWr_LW_ with seven significant associations, had the highest number of detected associations per trait. Two missense SNPs, MA_336364g0010_6123 and MA_64438g0010_10851 associated with TWr_LW,_ explained a small proportion of the PVE observed 0.01% and 0.03%, respectively. MA_336364g0010 is homologous to the *Arabidopsis INDUCER OF CBF EXPRESSION **2** (ICE2)* regulating deep-freezing tolerance by inducing *CBF1, CBF2* and *CBF3* genes (Table S1)^75^. *CBF* genes have been identified to constitute a central node of hormone cross-talk during cold stress response and their expression is modulated by abscisic acid, gibberelins, jasmonate, ethylene and brassinosteroids^76^. It has emerged that different hormone signaling pathways converge at the *CBF* promoter level, with the result of this hormone cross-talk being the fine-tuned transcript levels impacting on plant development and growth^77^. In spruce, the homolog of *ICE2* gene is highly expressed in developing stems (Fig. 2) and strongly upregulated in the cambium and radial expansion zone (Fig. 3) supporting its role in situ in promoting the tracheid expansion. Since *CBF*s have already been identified as convergence points for hormones required for the regulation of plant growth under cold stress, these factors would warrant a detailed look in relation to their influence on wood tracheid development, especially during the time when the water stress and cold stress can be common. The gene MA_64438g0010 is homologous to an *Arabidopsis PHOSPHATIDYLINOSITOL BINDING CLATHRIN ASSEMBLY PROTEIN 5B* (*PICALM5B),* a part of the ENTH/ANTH/VHS superfamily (Table S1). The ENTH/ANTH/VHS superfamily is involved in clatrin assembly at secretory vescicles and is essential for vescicle intracellular trafficking and thus, cell growth and development^78^. The gene was observed expressed in developing wood (Fig. 3), indicating its importance for tracheid development in spruce. Indeed, the genes of ENTH/ANTH/VHS family have been previously associated with secondary cell wall formation in *Populus*^26^, and vescicle trafficking-related genes were seen upregulated coinciding with radial expansion of developing wood cells in aspen^79^. Such genes are therefore expected to be associated with tracheid radial expansion in spruce. Another gene associated with TWr_LW_ was MA_950574g0010_7132, explaining a comparatively high PVE of 2.27%. It is remotely similar to *Arabidopsis CALCINEURIN-B-LIKE-INTERACTING SERINE/THREONINE-PROTEIN KINASE 23* (*CBLPK23*) involved in the regulation of HAK5-mediated high-affinity K^+^ uptake in calcium-dependent manner in *Arabidopsis* roots^80^. The confidence of the spruce model was low, but the gene was found highly expressed in developing shoots, buds and cones (Fig. 2), and during primary and secondary wall formation in developing spruce tracheids (Fig. 3) confirming that it was not a pseudogene. A *CALCINEURIN-B-LIKE* gene was found to explain the largest phenotypic variance in cell wall mannose content in white spruce^23^. These observations make the identified spruce *CBLPK23* gene an interesting candidate for calcium-dependent regulation of K^+^ uptake in developing tracheids, thus likely regulating tracheid expansion, similar to vessel element expansion, known to be dependent on K^+^ transport^81^. Interestingly, there was another candidate gene related to K^+^ transport associated with tracheid radial width: the splice variant MA_11172g0010_18275 explaining 0.01% PVE (Table 2). This gene is homologous to *Arabidopsis CYCLIC NUCLEOTIDE-GATED CHANNEL 17* (*CNGC17*) (Table S1). CNGCs are potassium channels involved in several plant physiological processes including root development, pollen tube growth and plant disease resistance^82^. They regulate ion homeostasis within plants through the uptake of cations, which is essential for plant growth and development^83^. *Arabidopsis* CNGC17 is localized in the plasmamembrane and promotes protoplast expansion by regulating cation uptake^84^. Its spruce homolog exhibited specific expression during latewood formation in August (Fig. 2), supporting its role in latewood tracheid development.

Three significant associations were identified for tangential tracheid width components with an upstream variant MA_10436040g0010_61320 being detected *across* traits TWt_TW_ and TWt_Ring_ (Table 2). This variant was detected on contig MA_10436040 with high inclusion frequencies explaining relatively high percentages of the variance observed, 2.13% for TWt_TW_ and 3.79% for TWt_Rin_ (Table 2). The associated gene—MA_10436040g0010—is homologous to the stress-related eukaryotic initiation factor 4A-III (eIF4A-III) which also has a DEAD-box ATP-dependent RNA helicase 2, and is involved in RNA processing and nonsense-mediated mRNA decay in *Arabidopsis*, especially under hypoxia and heat stress^85^ (Table S1). The spruce gene was not found expressed in available datasets (Fig. 2). SNP MA_10239556g0010_131776 was associated with TWt_EW_ and explained a moderate amount of the PVE 1.80% (Table 2). The *Arabidopsis* homolog encodes a subunit C of the vacuolar ATP synthase, which is a membrane-bound enzyme complex/ion transporter that combines ATP synthesis and/or hydrolysis with the transport of protons across the tonoplast membrane. This gene was highly and ubiquitously expressed (Fig. 2). All three SNPs were consistent with an additive mode of gene action (Table 2).

Twelve associations were detected for wall thickness components, with low to moderate PVE ranging from 0.01 to 1.78% (Table 2). Two of these associations (SNP MA_105586g0010_7132 and MA_10426383g0010_7358) were shared *across* cell wall thickness and coarseness traits. Ring average for cell Wall Thickness (WT_Ring_) had three significant associations. The synonymous SNP MA_492000g0010_1672 had a high inclusion frequency (0.726) and explained the highest percentage of PVE (1.78%). The same SNP was associated with WT_EW_. The gene *MA_492000g0010* is homologous to a tRNA synthetase beta subunit family protein, phenylalanyl-tRNA synthetase beta chain (Table S1). Consistent with its predicted general metabolic function in protein biosynthesis, it is ubiquitous and highly expressed in spruce tissues (Fig. 2). Missense SNP MA_9563494g0010_4010 and downstream variant MA_138164g0010_2032 explained 0.01% and 1.25% PVE, respectively. MA_9563494g0010_4010 is located in a gene *MA_9563494g0010* named as *Picea abies BIG GRAIN 2* (*PabBG2)*^83^ homologous to the *BIG GRAIN 1* gene (*OsaBG1*) in rice^87^. *OsaBG1* encodes a membrane protein regulating auxin transport and sensitivity, and positively affecting plant biomass and seed size. The gene belongs to a small family containing nine members in spruce^86^. Auxin has long been known to act as a key hormone essential for the induction of vascular strands, cambial growth and secondary wall deposition^88–91^. *PabBG2* is highly expressed and specifically upregulated in the developing xylem (Fig. 2) with a peak of expression in the cambial zone (Fig. 3), coinciding with a peak of IAA distribution in wood forming tissues^89,92^. It is therefore likely that the *PabBG2* gene pays a major role in xylogenesis, as suggested by its association with tracheid cell wall thickness, and that it should be considered as main target for woody biomass increase. Moreover, the SNP MA_138164g0010_78937 explaining PVE 1.25% associated with WT_Ring_ was located in a gene homologous to the subunit of E3 ubiquitin complex encoded by *AtAPC1* and involved in cell cycle regulation by degradation of cyclin B1^93^. The E3 ubiquitin complex is also known in *Arabidopsis* to regulate auxin homeostasis^93–95^. Hence, the detection of two significant associations for WT_Ring_ that are potentially related to auxin regulation implies a close relation between auxin and cell wall thickness in spruce. A QTL in rice grain for width and weight, which is related to plant biomass, has been associated with a RING-type E3 ubiquitin ligase^97^. Several auxin responsive genes were also associated with tracheid width and MFA, which both are linked to cell wall thickness, in white spruce^23^.

WT_EW_ has three significant associations beside MA_492000g0010_103329 discussed above (Table 2). The missense variant MA_80033g0010_51296 was within a gene encoding a MYB transcription factor similar to *Arabidopsis* MYB68 (Table S1). This gene exhibited very low expression levels in the developing xylem but rather was expressed in young shoots and needles (Fig. 3). Different MYB transcription factors regulate plant developmental processes, and several have been identified as crucial factors for secondary wall deposition and lignification. Loblolly pine (*Pinus teada* L.) *PtMYB8* expressed in spruce induced secondary cell wall thickening^98^. White spruce (*P. glauca* L.) *PgMYB4* was associated with cell wall thickness and tracheid coarseness^23^, and has been shown to be highly expressed during secondary cell wall formation and lignification in both white spruce and loblolly pine^99^. MYB encoded by *MA_80033g0010* could play a more indirect role in secondary wall regulation in spruce considering its expression (Fig. 2). Two remaining SNPs MA_17843g0010_19482 and MA_105586g0010_7132, had PVEs of 0.01% and 0.10%, respectively (Table 2). The former was a missense variant within a gene homologous to *Arabidopsis TOC64-V*. The latter was not matching any known gene and was also associated with C_EW_ and explaining a moderate percentage of PVE 2.08%. However, the two models were not expressed in any of the previously reported spruce expression studies (Fig. 2).

WT_LW_ was associated with four upstream variants and a single synonymous SNP MA_10426383g0010_7358. The four upstream variants explained PVE ranging from 0.01 to 0.10% whereas the synonymous SNP MA_10426383g0010_7358 had a high inclusion frequency and explained a moderate amount of the PVE 1.57% (Table 2). *MA_10426383g0010* is homologous to *VIT_16s0098g01810* from *Vitis vinifera* (Table S1) annotated as encoding ATP binding protein that may be involved in chromosome organization and biogenesis^97^. The *Arabidopsis* homolog—*GAMMA-IRRADIATION AND MITOMYCIN C INDUCED 1* (*GMI1*) is responsible for double strand repair via somatic homologous recombination^100^. The spruce gene shows increased expression in organs with active meristems (Fig. 2), which is expected for the function in DNA repair. The same SNP MA_10426383g0010_7358 was also associated with traits related to coarseness (C_TW_ and C_LW_) and explained a relatively high PVE of 3.25% and 1.40%, respectively. It also had high inclusion frequencies for all three traits (WT_LW_, C_TW_ and C_LW_) (Table 2). The associated gene might therefore be a good candidate to explore for effects on tracheid development, especially since it is highly expressed in the developing wood^74^ (Fig. 2). SNP MA_5g0010_1 associated with WT_LW_ was detected upstream of gene *MA_5g0010* belonging to the 4-coumarate-CoA ligase (4CL) family, which includes key enzymes in the monolignol biosynthetic pathway. However, the *Arabidopsis* homolog of *MA_5g0010, At4g05160* does not encode an enzyme active on phenyl propanoid substrates but a fatty acyl CoA synthase involved in lipid and jasmonic acid biosynthesis^101^. *MA_5g0010* is not expressed in the developing wood but it is highly expressed in young vegetative shoots and needles, including the infected needles (Fig. 2), making it an unlikely candidate for lignin biosynthesis in developing wood but suggesting a rather indirect function in the regulation of tracheid cell wall thickness. The SNP MA_9125g0010_34791 associated with WT_LW_ was located upstream of a gene homologous to *Arabidopsis OBERON2 (OBE2)* encoding a plant homeodomain (PHD) finger protein (Table S1) (Lee et al*.* 2009). Homeodomain genes encode transcription factors central in the regulation of plant developmental processes^102^. *OBE1* and *OBE2* redundanlty regulate meristem establishment and maintenance in *Arabidopsis* (Saiga et al*.* 2008). The spruce *OBE2* gene is ubiquitous and highly expressed in vegetative and reproductive organs (Fig. 2) including developing wood where it shows high expression during secondary wall deposition (Fig. 3) and therefore it could have a direct role in cell wall thickening in tracheids. SNP MA_885527g0010_112677 associated with WT_LW_ was found upstream of a gene containing a SET domain. SET domain proteins have been identified in *Arabidopsis* to play aide in the epigenetic control of genes involved in a wide range of activities including plant growth^96^. A link has also been established between PHD finger proteins and SET domain proteins in the regulation of developmental transitions in *Arabidopsis* where PHD finger proteins VEL1, VRN5 and VIN3 interacting with H3K27me3 repress FLC transcription allowing for the transition from vegetative to reproductive development^103^. *MA_885527g0010* is highly upregulated in developing wood from August that is involved in latewood biosynthesis (Fig. 2) suggesting its direct role in latewood tracheid development.

A total of five significant associations were identified for coarseness traits explaining moderate to high PVE ranging from 0.78 to 3.62% (Table 2). Two of them, SNPs MA_105586g0010_7132 and MA_10426383g0010_7358 were also associated with WT_EW_ and WT_LW_, and discussed above. An Upstream variant MA_373300g0010_1844 associated with C_TW_ explained a relatively high percentage of PVE 3.62% and was consistent with a partial to fully dominant mode of gene action (Table 2) as shown by the genotypic effects (Fig. 1). The gene is similar to *Potri.T064000* from *Populus trichocarpa* annotated as encoding a protein kinase similar to wall-associated receptor kinase-like (WAK-like) proteins. WAKs have been previously reported to be associated with average ring width and the proportion of earlywood in white spruce^23^ and with MFA in *Populus*^26^. The gene is expressed primarily in early season needles and late season stem from vegetative shoots but there is no detectable expression in developing xylem (Fig. 2), suggesting its indirect involvement in the regulation of tracheid coarseness.

## Conclusion

This work presents the first genome wide dissection of wood tracheid traits in Norway spruce. A total of 30 significant associations were detected for all investigated traits. These associations have identified a set of genes that could be exploited to alter wood tracheid traits for improving solid wood properties for its use in industrial processes. Previous studies utilizing a LASSO penalized analysis approach were limited in the nature and number of molecular markers available^28,30^, with our study representing a major advance by using 178,101 SNPs with a functional mapping approach. The relatively small number of associations is comparable to other association studies of complex growth traits in forest trees, were a few associations are detected with a relatively small proportion of the genetic variation being explained^26,27,104–106^. It can be argued that many of the alleles causing variation for polygenic traits may be either rare or have small effects and current GWAS methods lack the power to detect them, thus the small number of significant associations^107,108^. The small number of associations being reported could also be largely due to the small sample sizes in these studies for such complex traits. Theoretical work has also shown that alleles of large effect are unusual, with allele effect having been suggested to follow a negative exponential distribution pattern^109^. Thus the magnitude of the detected allele effects follows a truncated exponential distribution^110^. Therefore, the detection of alleles with small effects is difficult when compounded with the small population size. The small number of significant associations can also be attributed to the genotyping method, which is a complexitiy reduction genotyping method. The limitation of the genotyping employed in our study has also been noted in other studies^111^, in that some of the alleles impacting a trait might not be within the captured regions that we targeted. If the sampled markers do not include the casual allele or if the LD between the marker and the casual allele is incomplete the power of detection is drastically reduced^26^. The statistical power required to detect associations between molecular markers and a trait is heavily dependent upon the sample size^112,113^. Due to the challenges of developing large populations for GWAS in conifers, the majority of the studies utilize a few hundred individuals from natural populations, which limits the statistical power of GWAS. It was reported that in order to capture 50% of genetic variaon for growth traits in an association mapping study, it would require roughly 25,000 individuals to be analysied^14^. Therefore, the relatively small association population size results in low statistical power, thus rendering small to medium effect QTLs statistically non-significant and very difficult to detect. Our study had 517 martenal trees to perform GWAS upon, thus rendering a small number of significant associations. Missing heritability will remain an issue in association studies as long as population sizes are kept in the range of hundreds^14^. However, improvements made to statitstical methods are now potential viable options, which are being developed and utilize a combination of information from multiple populations using Meta-GWAS and Joint-GWAS^114,115^. These approaches are now being applied in some recent forest tree studies^113^ and could be the next level of analysis using our application of latent traits on these complex traits.

## Supplementary information

Supplementary file1

## Data Availability

All the latent traits, genotypic data, SNP position files the association mapping scripts used for the analysis are publicly available at are available from zenodo.org at https://doi.org/10.5281/zenodo.3374415 (https://doi.org/10.5281/zenodo.3374414) . Raw sequence data for all the samples utilized in the study are found through the European Nucleotide Archive under accession number PRJEB29652. The Norway spruce genome assemblies and resources are available from https://congenie.org/pabiesgenome.
